# Moral reframing of messages about mask-wearing during the COVID-19 pandemic

**DOI:** 10.1038/s41598-023-37075-3

**Published:** 2023-06-22

**Authors:** Jonas T. Kaplan, Anthony Vaccaro, Max Henning, Leonardo Christov-Moore

**Affiliations:** 1 Brain and Creativity Institute and Department of Psychology, University of Southern California, Los Angeles, USA; 2 Present Address: Institute for Advanced Consciousness Studies, Santa Monica, USA

**Keywords:** Human behaviour, Patient education

## Abstract

When communicating about political issues, messages targeted to resonate with the core values of the receiver may be effective, an approach known as *moral reframing*. During the COVID-19 pandemic, we tested the relationships between moral values and mask-wearing in a sample (N = 540) of self-identified liberals, conservatives, and moderates in the United States. Anti-mask attitudes were stronger in conservatives, and were associated with increased concerns for in-group loyalty, national identity, and personal liberty. We then crafted messages about the benefits of mask-wearing framed to resonate with these moral concerns, and in a pre-registered study of N = 597 self-identified U.S. conservatives, tested the effect of moral reframing on anti-mask attitudes and behaviors. Messages framed in terms of loyalty, with appeals to the protection of the community and America, were effective in reducing anti-mask beliefs, compared with unrelated control messages and messages delivering purely scientific information, and these changes in belief persisted for at least 1 week. Exploratory analyses showed that participants who saw loyalty-framed messages reported wearing masks in public more frequently in the subsequent week. This study provides evidence that framing messages about health behaviors in terms of group loyalty may be one productive way of communicating with conservative audiences.

## Introduction

Despite strong evidence that facemasks are an effective tool in reducing the transmission of SARS-CoV-2^1–12^, Americans have been divided in their reactions to public policies that recommend mask-wearing. A poll by Pew Research in June 2020 found that 63% of Democrats agreed that masks should always be worn in public compared with only 29% of Republicans^13^, and several recent studies have found that increased conservatism is associated with reduced intention to wear masks^14–16^. Conservatives also engage in less social distancing^17^ and perceive COVID-19 as less threatening^18^ compared with liberals. Thus, mask-wearing in America appears to have become politicized.

In the process of politicization, a belief becomes associated with a collective political identity, and is therefore tied to an adversarial ingroup/outgroup conflict^15,19,20^. Politicization may entail the *moralization* of political beliefs, in which a political position changes from a utilitarian means to an end into a matter of right and wrong^21–23^. When a preference becomes a moralized attitude, people become less likely to compromise, more motivated to fight for what they believe is right, and more likely to experience strong emotions in relation to the belief^22,24–27^. Once there is a moral imperative involved, an issue tends to be viewed in absolutes and people view their moralized attitudes as grounded in unchallengeable objective truths^26^.

Moralized beliefs, then, are likely to be more difficult to change. Research on the correction of misinformation has yielded mixed results, and it is not yet well understood why information is sometimes effective and other times ineffective. While corrective information can sometimes change beliefs^28^, often the effects of misinformation persist beyond correction^29–32^, and beliefs associated with moral convictions and with political identities may be especially resistant to revision^33–36^. Therefore, understanding the moral values that underlie the targets of health messages is crucial^37^. Since American conservatives tend to be less trusting of science compared with liberals^38,39^, the communication of scientific information is especially challenging with this population^40^. Indeed, recent studies have found that low trust in science is associated with belief in misinformation about COVID-19^41^ and with anti-mask attitudes specifically^14^.

One approach to communicating about moralized beliefs that shows promise is *moral reframing*. In moral reframing, messages are framed to resonate with the moral values of the listener^42,43^. Persuasive appeals framed in moral terms are more effective when communicating about moralized issues^44^, and work better when the value frame of the persuasive message matches the values of the receiver^45–47^. One framework that has been used to study the differences between the moral concerns of liberals and conservatives is the Moral Foundations Theory (MFT), which describes five moral domains of concern: care/harm, fairness/cheating, loyalty/betrayal, authority/subversion, and sanctity/degradation^48^. Notably, conservatives tend to show stronger concern compared with liberals for moral values related to loyalty, authority, and sanctity^48^. In one example of successful moral reframing, studies have found that conservatives are more receptive to environmental appeals that are framed in terms of sanctity, for example by describing the pollution or contamination of the natural environment^49,50^.

If the issue of mask-wearing has become politicized, there is likely to be a connection between attitudes towards mask-wearing and underlying moral concerns. In a first study, we aimed to characterize the relationships between beliefs and attitudes surrounding mask-wearing and the moral concerns of people who hold those beliefs. Given the association between anti-mask attitudes and conservatism, we expected to find an association between conservative beliefs and the moral concerns of loyalty, authority, and purity in a sample of subjects from the United States. In a second pre-registered study, we crafted messages that used moral reframing to communicate about the benefits of mask-wearing, targeting the core moral values shared by people who hold strong anti-mask beliefs. We hypothesized that messages whose framing matched the moral concerns of receivers would lead to greater belief and behavior change compared with messages that do not match, or those that contained only scientific information with no moral framing.

### Study 1: the association of moral concerns with mask-related beliefs

In this first exploratory study we aimed to characterize the relationships between beliefs about mask wearing during COVID and of the people who hold these beliefs, including their moral concerns, their trust in science, identification with community, authoritarianism, and attitudes towards COVID and mask-wearing in general.

## Methods

In this study, we report all measures, manipulations and exclusions. Some survey measures are described but were not included in our analysis as they were not relevant to the hypotheses explored here. Those measures are detailed below.

### Participants

 Participants were recruited online using Prolific (http://www.prolific.co). All subjects resided in the United States and were over 18 years of age. We aimed to enroll 540 subjects, 180 in each of three groups: liberals, moderates, and conservatives, based on their self-identified political orientation as provided by Prolific. We ultimately received responses from 181 Liberals, 182 Moderates, and 179 Conservatives. All survey responses were completed on the day of June 11, 2020. Median time to complete the survey was 15 min and 11 s. After removing incomplete responses (defined as more than one item unanswered in any of the measures), we were left with 179 Liberals, 182 Moderates, and 172 Conservatives for a total of N = 533. Participants were 51% female, 48% male, with 1% preferring not to describe their gender, and ranged in age from 18 to 84 years (M = 33.1, SD = 13.39).

### Procedure

 All study procedures were approved by the Institutional Review Board of the University of Southern California and were performed in accordance with all relevant ethics guidelines and regulations. Informed consent was obtained from research subjects prior to their participation. Participants completed on online survey using the Qualtrics software (http://www.qualtrics.com) to complete the following measures: The Moral Foundations Questionnaire, the Trust in Science and Scientists Inventory, the Identification With All Humanity Scale, the Very Short Authoritarianism Scale, a 16-item questionnaire assessing Anti-mask beliefs, a 12-item scale assessing adherence to COVID guidelines, and a question asking about reasons for not wearing masks.

### Survey measures

There were several demographic measures collected that are not reported here as they were not relevant to our hypotheses. These include education, religiousness, age, SES, and recent voting behavior. All other measures are reported here.

#### Moral Foundations Questionnaire

 The short version of the Moral Foundations Questionnaire (MFQ20) is a 20 question self-report measure which assesses the degree to which individuals prioritize five moral domains in their decision-making^48^. Subscales of the MFQ include: Harm/Care (alpha = 0.76), Fairness/Reciprocity (alpha = 0.76), Ingroup/Loyalty (alpha = 0.68), Authority/Respect (alpha = 0.76), and Purity/Sanctity (alpha = 0.79). In Part 1 of the MFQ, respondents indicate^48^ the relevance of various situational considerations to their decision-making about moral right or wrong using a six point scale from “not at all relevant” to “extremely relevant”. In Part 2 respondents indicate their agreement or disagreement with a series of moral statements, using a six-point scale from “strongly disagree” to “strongly agree”.

#### Trust in Science and Scientists Inventory

 The Trust in Science and Scientists Inventory is a 21 question self-report measure which assesses an individual's domain-general level of trust in science and scientists^51^. Questions assess trust in science in two areas: (1) perceptions of the nature of science, specifically the tentative nature of scientific knowledge, and (2) perceptions of certain research processes or scientist integrity. Respondents rank their level of agreement with a series of statements of trust in science and scientists in the two areas noted above, using a five-point scale from “strongly disagree” to “strongly agree”. Internal consistency for this scale was high (alpha = 0.93).

#### Identification With All Humanity (America/Community/All humanity)

 The Identification With All Humanity (IWAH) is a nine-question self-report measure which assesses an individual's concern for the interests and concerns of all people^52^. The IWAH has been shown to measure a construct which is not simply the absence of ethnocentrism and its correlates, and is more than the presence of empathy, general morality, universalism, and principled moral reasoning alone. Each of the 9 questions assess respondent identification with (1) “People in my community” (alpha = 0.85), (2) “Americans” (alpha = 0.83), and (4) “People all over the world” (alpha = 0.80). Respondents answer on a five-point scale from 1 (not at all) to 5 (very much).

#### Very Short Authoritarianism Scale

 The Very Short Authoritarianism (VSA) Scale is a six-item self-report measure that is a shortened version of Altemeyer’s Right Wing Authoritarianism (RWA) scale^53^. The RWA assesses three subdimensions of RWA: Authoritarian Submission, Authoritarian Aggression, and Conventionalism. The VSA was designed to assess the overall RWA construct, equally representing the three content subdimensions of the RWA while also balancing the direction of wording effects within these subdimensions as in the Authoritarianism Conservativism Traditionalism (ACT) scale^54^. Respondents indicate agreement/disagreement with statements on a nine-point scale from “unsure/neutral” to “very strongly agree/disagree”. Internal consistency for this scale was high (alpha = 0.81).

#### Anti-mask Belief (16 items)

 The Anti-Mask Belief scale was constructed of 16 questions reflecting common sentiments expressed by individuals who advocate against mask-wearing, as well as beliefs they do not endorse which are shared by the scientific community and those for the use of masks. 14 of the questions were responded to on a 7-point scale from “Strongly Disagree” to “Strongly Agree”. The remaining two questions (“How effective do you believe wearing a mask is at protecting yourself from COVID-19?” and “How effective do you believe wearing a mask is at preventing you from transmitting COVID-19 to others?”) were responded to on a 7-point scale from “Not effective at all” to “Extremely effective”. Internal consistency for this measure was high (alpha = 0.87). The questions for this scale can be found in the below.

#### COVID Guideline Adherence (12 items)

 COVID Guideline Adherence consisted of 12 questions related to how often participants followed current health guidelines on preventing the spread of COVID-19 (social distancing, hand-washing, etc.). Questions were responded to on a 5 point scale from “Not at all” to “Always”. Internal consistency for this measure was high (alpha = 0.89). The questions for this scale can be found below.

#### Mask-wearing reasons

Participants were asked to click a check box next to any of several reasons why they might not wear a mask. Those reasons are listed below.

#### Data analysis

 Data were analyzed using R version 4.0.1^55^. Analysis code and data are available on the OSF website for this study. We first computed a simultaneous ordinary least squares linear regression model predicting Anti-mask Belief as a function of the 12 other measures. This model was conducted using the lm function in R as follows:

$$\begin{aligned} \left[ {{\text{Antimask }}\;{\text{Belief}}} \right] & \sim \left[ {{\text{Conservatism}}} \right] \, + \, \left[ {{\text{Trust In }}\;{\text{Science}}} \right] \, \\ & \;\; + \, \left[ {{\text{Authoritarianism}}} \right] \, + \, \left[ {{\text{MFQ }}\;{\text{Harm}}/{\text{Care}}} \right] \, + \, \left[ {{\text{MFQ }}\;{\text{Fairness}}} \right] \, \\ & \;\; + \, \left[ {\text{MFQ Loyalty}} \right] \, + \, \left[ {{\text{MFQ }}\;{\text{Purity}}} \right] \, + \, \left[ {{\text{MFQ}}\;{\text{ Authority}}} \right] \, + \, \left[ {{\text{Identification }}\;{\text{with}}\;{\text{ America}}} \right] \, \\ & \;\; + \, \left[ {{\text{Identification }}\;{\text{With }}\;{\text{Community}}} \right] \, + \, \left[ {{\text{Identification}}\;{\text{ With}}\;{\text{ All }}\;{\text{Humanity}}} \right] \\ \end{aligned}$$

A sensitivity power analysis computed with G*Power shows that this linear model with N = 533 and 12 predictors at α = 0.05 has 80% power to find an effect size of Cohen’s *f* = 0.18. To test for political group differences in mask-related belief and attitudes towards COVID, one-way independent samples ANOVAs were used to compare Anti-mask Belief and COVID Guideline Adherence across liberals, conservatives, and moderates. A sensitivity power analysis shows that this 3-way ANOVA with α = 0.05 has 80% power to find an effect size of partial η^2^ = 0.18. Pairwise post-hoc comparisons between groups were performed using the Tukey HSD test.

Next, we computed Pearson correlations among 13 measures of interest. A sensitivity power analysis finds that each correlation tested with at α = 0.05 has 80% power to find an effect size of *r* = 0.12. We corrected for multiple comparisons using a Bonferroni correction across the 78 unique pairwise correlations.

We then computed the proportion of respondents that checked each of the reasons for not wearing a mask and computed a chi-square test to compare the frequencies across the three political groups for each question. A sensitivity power analysis for this test with α = 0.05 has 80% power to find an effect size of *w* = 0.13. Each question was tested separately and a Bonferroni correction was applied to control for multiple comparisons across the 12 items.

## Results

### Correlations among measures

 These are reported in the Supplemental in Table S1 and depicted in Fig. S2.

### Group comparisons

**Figure 1:**
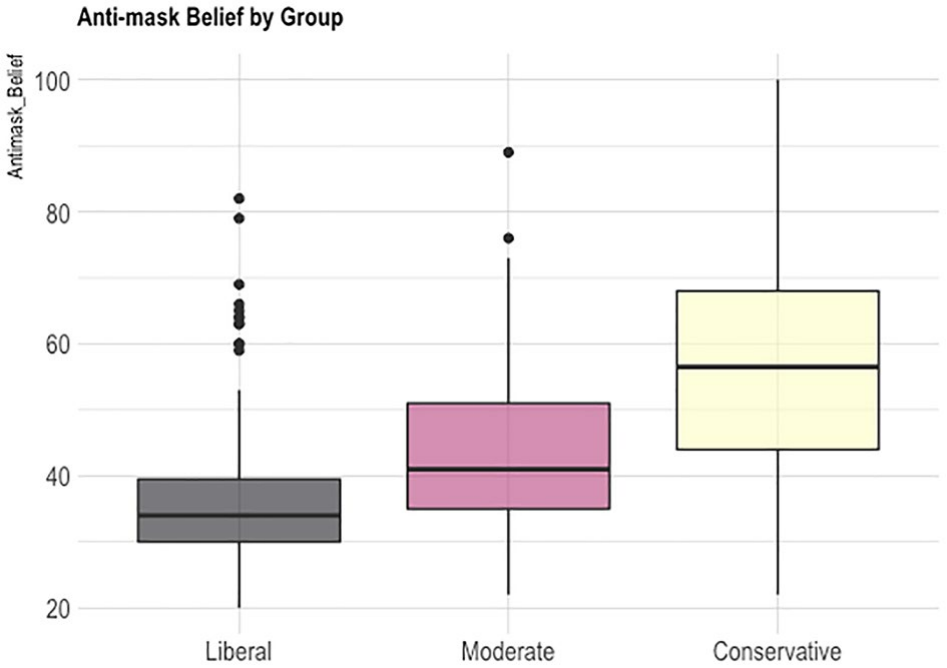
Anti-mask belief by group in study 1.

**Figure 2:**
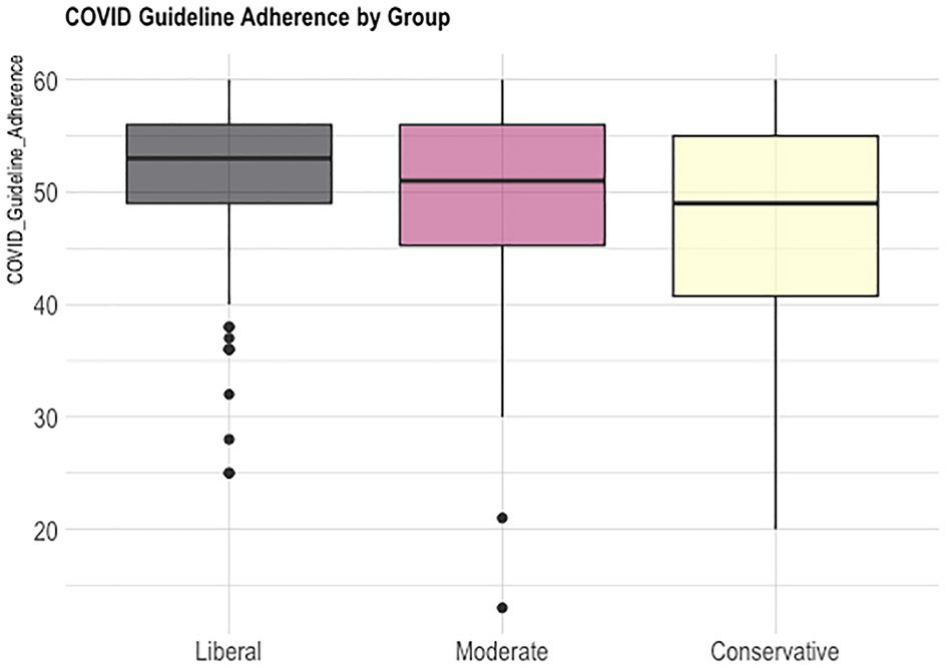
Reported COVID guideline adherence by group in study 1.

 There was a significant main effect of political group for Anti-mask Belief (F(2,530) = 99.04, p < 0.001, partial η^2^ = 0.272), and post-hoc Tukey HSD tests revealed greater Anti-mask Belief for Conservatives (M = 56.3, SD = 16.8) compared to Moderates (M = 43.6 SD = 12), for Moderates compared to Liberals (M = 36.5, SD = 10.5), and for Conservatives compared to Liberals (see Fig. 1). There was also a significant main effect of political group for COVID Guideline Adherence (F(2,530) = 13.14, p < 0.001, partial η^2^ = 0.047), with the Tukey test showing Conservatives (M = 47.0, SD = 9.95) with lower adherence compared to Moderates (M = 49.6, SD = 7.99), and compared with Liberals (M = 51.5, SD = 6.86) (see Fig. 2).

### Regression

 Full regression results are presented in table S2. As a test for multicollinearity, Table S3 presents the variance inflation factor (VIF) for each variable in the mode. The highest VIF was Authority/Respect at 4.04. The regression model significantly predicted Anti-mask Belief (F(10,521) = 46.02, p < 0.001), with an R^2^ of 0.492 (Cohen’s *f* = 0.99). Trust In Science inversely predicted Anti-mask Belief (β = − 0.53, t(521) = − 12.351; 95% CI = − 0.6168, − 0.4475; p < 0.001; *f* = 0.29), as did MFQ Harm/Care (β = − 0.680, t(521) =  − 3.476; 95% CI = − 1.0645, − 0.2958; p < 0.001; *f* = 0.02) and MFQ Fairness/Reciprocity (β = − 0.503, t(521) =  − 2.62; 95% CI = − 0.8811, − 0.1253; p < 0.001; *f* = 0.01), and Identification with All Humanity (β = − 0.204, t(521) = − 2.163; 95% CI = − 0.3892, − 0.0187; p = 0.031; *f* = 0.01). Two variables positively predicted Anti-mask belief: MFQ Ingroup/Loyalty (β = 0.555, t(521) = 2.99; 95% CI = 0.1899, 0.9193; p = 0.003; *f* = 0.02) and Identification With America (β = 0.334, t(521) = 3.077; 95% CI = 0.1208, 0.5479; p = 0.002; *f* = 0.02). Conservatism (β = 0.16, t(521) = 0.745, p = 0.456), Authoritarianism (β = − 0.02, t(521) = 0.69, p = 0.766), MFQ Authority/Respect (β = − 0.02, t(521) = − 0.1, p = 0.92), MFQ Purity/Sanctity (β = − 0.2, t(521) = − 0.13, p = 0.89) and Identification with Community (β = − 0.1, t(521) = 0.08, p = 0.242) were not significant predictors of Anti-mask Belief.

### Reasons for not wearing masks

 The proportion of respondents selecting each reason for not wearing masks is shown in Table 1. There were 7 items that showed significant differences across the political groups. All of these differences involved conservatives endorsing the reason more frequently than the other groups. The most common reason for not wearing masks cited among conservatives was “Because it’s my choice not to wear one” (34.9% of conservatives, 14.2% of moderates, and 3.4% of liberals chose this reason), and the second-most common reason was “Because masks are uncomfortable” (30.2% of conservatives, 15.9% of moderates, and 8.9% of liberals chose this reason).

**Table 1:** Proportion of respondents endorsing each reason for not wearing a mask.

Reason	Liberals	Moderates	Conservatives	X^2^	Effect size (*w*)	*p*	Significant
Because masks are uncomfortable	0.089	0.159	0.302	27.669	0.23	9.81E−07	*
Because I don’t think it will prevent me from getting sick	0.039	0.077	0.238	38.057	0.27	5.45E−09	*
Because I don’t think it will prevent others from getting sick	0.022	0.06	0.157	23.185	0.21	9.23E−06	*
Because it looks silly	0.011	0.033	0.116	31.594	0.24	2.05E−05	*
Because it's my choice not to wear one	0.034	0.142	0.349	62.779	0.34	2.33E−14	*
Because it looks weak	0.017	0	0.087	23.001	0.21	1.01E−05	*
Because I don’t have one	0.101	0.093	0.11	0.284	0.02	8.68E−01	
Because it fogs up my glasses	0.084	0.093	0.145	4.009	0.09	1.35E−01	
Because my friends aren’t wearing them	0.039	0.033	0.041	0.165	0.02	9.21E−01	
Because it is socially awkward to wear one	0.039	0.033	0.076	4.005	0.09	1.35E−01	
Because it is my right not to wear one	0.011	0.088	0.186	31.667	0.24	1.33E−07	*
Because it's hard to exercise while wearing a mask	0.122	0.159	0.198	3.658	0.08	1.61E−01	

## Discussion

First, conservatives in our sample did indeed harbor stronger beliefs against mask-wearing compared with liberals, with moderates showing a level of endorsement of these beliefs somewhere between conservatives and liberals. Conservatives also reported complying less with COVID-related guidelines more generally. This is consistent with polling and with recent research showing political polarization of mask wearing and other social distancing measures and supports the observation that issues surrounding the pandemic have become politicized.

Importantly, we found that beliefs against mask wearing were associated with people’s underlying moral concerns. Specifically, in our regression model, concerns for Harm and Fairness were associated with lower anti-mask belief, while concerns for Loyalty were associated with increased anti-mask belief. Interestingly, these loyalty concerns appear to be directed at the level of national identity; stronger Identification with America predicted stronger anti-mask beliefs, while Identification with Community did not. Notably, Identification with America predicted ant-mask belief even when controlling for Loyalty.

Conservatives’ most commonly reported reason for not wearing masks were that “it’s my choice not to wear one” and they were over 10 times more likely than liberals to report this reason for not wearing a mask. Conservatives also were likely than liberals to say that they don’t wear masks because “it is my right not to wear one”. This result suggests that a concern for personal liberty may be at play for conservatives when thinking about the issue of mask wearing. The version of the MFQ that we used did not measure the value axis of Liberty/Oppression, which has been hypothesized as a sixth moral foundation^56^, and it is not clear to what extent conservatives in general are more concerned with libertarian values.

### Study 2: moral reframing of messages

The second study was designed to test if moral reframing of messages about mask wearing, targeted to resonate with the values of conservatives, would be more effective at changing beliefs and behaviors, compared to messages presented in a purely science-based framing, or compared to a control video which contained no information about the benefits of mask wearing. Study 1 established that conservatives in our sample did indeed tend to report less mask wearing and greater anti-mask belief. Study 1 also identified two promising values that could be used to reframe mask-wearing messages: (1) ingroup/loyalty, with a specific focus on identification with America, and (2) personal liberty. Ingroup/loyalty as measured by the MFQ was predictive of anti-mask belief, and reported reasons for avoiding masks suggested a concern for personal liberty (which was not measured by the version of the MFQ we used in Study 1). While Fairness and Harm also predicted these beliefs, people who scored high on these values were *more* likely to wear masks, making these values poor targets for message framing. In short, the results of Study 1 suggested that messages framed in terms of loyalty and liberty could appeal to conservative audiences.

We pre-registered this study at the Open Science Foundation: https://osf.io/kbzd4/?view_only=3e1b1c41a5304dc49ff26074c156af5b. Participants in this study watched a short video online, then answered questions about their mask-related beliefs immediately following the video and again, one week later.

We sought to test three hypotheses. H1: *Scientific information about the benefits of mask wearing would not increase intention to wear masks and would not decrease anti-mask belief;.* H2: *Moral reframing of the benefits of wearing masks in terms of liberty or loyalty would be more effective than the control condition*; H3: *Combining moral reframing with scientific information would be more effective than both scientific information alone and moral reframing alone*.

## Methods

In this study, we report all measures, manipulations and exclusions.

### Participants

 Participants were recruited through Prolific (https://www.prolific.co/) and were directed to a Qualtrics survey that presents the stimuli and collects the responses. The participants targeted through Prolific’s sample curating self-identified as conservative and currently reside in the United States. Self-identified political orientation was made by Prolific’s prescreening questionnaire in which participants answer optional questions about their background. For political orientation, potential subjects choose liberal, moderate, or conservative to describe themselves. Participants were paid $3 per session for participating, upon completion of the two sessions, one week apart. To reach our target of 800 participants, or 160 in each of 5 groups, we recruited 848 people, of whom 760 returned a week later to complete the second part of the study. The rationale for the sample size and a power analysis is detailed in the pre-registration documents. Details on the exclusion of subjects can be found in the Supplemental. The final group of 597 subjects had an age range of 18–77 (M = 39.17, SD = 14.18), 259 identified as female, 336 as male, and 2 preferred not to describe their gender. Median time to complete the first session was 9 min, 8 s, and median time to complete the second session was 6 min, 15 s.

### Procedure

 We manipulated the type of health message received by the participants by presenting each group with a different short video. Each video was about 3 min long. Each video presented 8–10 “scenes” which consisted of photos or videos with text in front of them. The “combined” condition, in order to effectively include elements of the other conditions, was longer than the others and contains 13 scenes. In a between-subjects design, each participant was randomly assigned to one of the 5 conditions.

Detailed descriptions of the video content are in the Supplemental, links to the videos on YouTube are in Appendix 1, and the text of the videos is found in Appendix 2. In brief, there was a CONTROL video with no information about mask-wearing, SCIENCE, LIBERTY, LOYALTY videos with information framed accordingly, and a COMBINED video that included elements of all three frames. To validate the moral content of the stimuli, six experts in the field who were familiar with the MFT and were not previously familiar with the stimuli rated the videos for each of the related moral concerns. For each morally framed video the intended moral concern was the highest rated, although notably the Liberty video was found to contain substantial reference to Loyalty as well. Full results of this analysis can be found in the Supplemental Materials.

Because of subject exclusions, the final number of subjects in each group was not equal. There were 130 subjects in the CONTROL condition, 110 in the SCIENCE condition, 112 in the LOYALTY condition, 121 in the LIBERTY condition, and 124 in the COMBINED condition.

The survey at T1 contained measures of basic demographics, political orientation, mask wearing behavior, anti-mask belief, and reaction to the videos. The main dependent measures were mask-wearing behavior and anti-mask belief. Additional variables of interest that were modeled in the analysis include: conservatism, gender, and age. The survey at T2 repeated the measures of mask wearing behavior and anti-mask belief, and added COVID threat perception, and COVID health behavior compliance. At T2 we asked about mask-wearing behavior during the week between the two measures. At T1, we did not ask about mask-wearing behavior during the week prior to T1 because (a) we wanted participants to watch the videos without having been primed to think about mask-wearing and (b) if we asked after the video, retrospective responses could be affected by having seen the video.

### Survey measures

**Anti-mask Belief** (alpha = 0.91): This is the same scale described in Study 1.

**COVID risk perception** (alpha = 0.91): This is the same scale described in Study 1.

**Video attention check questions:** After watching the video, participants answered two questions about the content of the specific video that they saw.

**Video reaction questions.** Participants were asked to rate on a scale from 1 to 5 where 1 was “not at all” and 5 was “extremely”, to what extent they found the video to have each of the following qualities: compelling, informative, interesting, enjoyable.

### Data analysis

#### Pre-registered analyses

As described in the pre-registration, data were analyzed using the general linear model, with a simultaneous multiple linear regression approach. First, we modeled the dependent variables from T1: Anti-mask belief at T1 will be modeled as a linear function of: video type, age, gender, and political orientation, using the lm function in R as follows:

$$\left[ {{\text{Anti}} - {\text{mask Belief}}} \right] \, \sim \, \left[ {\text{Video type}} \right] \, + \, \left[ {{\text{Gender}}} \right] \, + \, \left[ {{\text{Conservatism}}} \right] \, + \, \left[ {{\text{Age}}} \right]$$

Video type is a categorical variable with 5 levels that were dummy coded. A sensitivity analysis of this model with α = 0.05 finds that it has 80% power to find an effect size of *f* = 0.15. An ANOVA was also performed for the main effect of video type using the aov function in R. Planned contrasts compared each video to baseline (control) using the Dunnett procedure to test H1 and H2. To test H3, the Combined condition was be compared to the Liberty, Loyalty, and Science conditions using a contrast weighted [0 -1 -1 -1 3] for Control, Science, Liberty, Loyalty, and Combined). For data from T2, the same procedure as T1 as be employed, but at T2 there are two dependent variables that were modeled separately: Anti-mask belief, and mask-wearing behavior.

#### Exploratory analysis

 At the end of the T1 experiment, participants reported how compelling, interesting, informative, and enjoyable they found the video. This reaction may be an important predictor of the effectiveness of the video but was not included in our pre-registered design. In an exploratory analysis, we re-computed each model including a measure of reaction to the videos. Since these 4 ratings were highly intercorrelated (α = 0.93), we averaged the ratings into one video reaction judgment. The regression model in R was computed with lm as follows, using the dependent Anti-mask belief as an example:

$$\left[ {{\text{Anti - mask}}\;{\text{ Belief}}} \right] \, \sim \, \left[ {{\text{Video}}\;{\text{ type}}} \right] \, + \, \left[ {{\text{Gender}}} \right] \, + \, \left[ {{\text{Conservatism}}} \right] \, + \, \left[ {{\text{Age}}} \right] \, + \, \left[ {{\text{Video}}\;{\text{ reaction}}} \right]$$

Because these analyses were not pre-registered, and to account for multiple comparisons (5 models tested), we used a stricter alpha threshold of p = 0.01.

## Results

### Pre-registered analyses

#### Tests of H1 and H2

**Figure 3:**
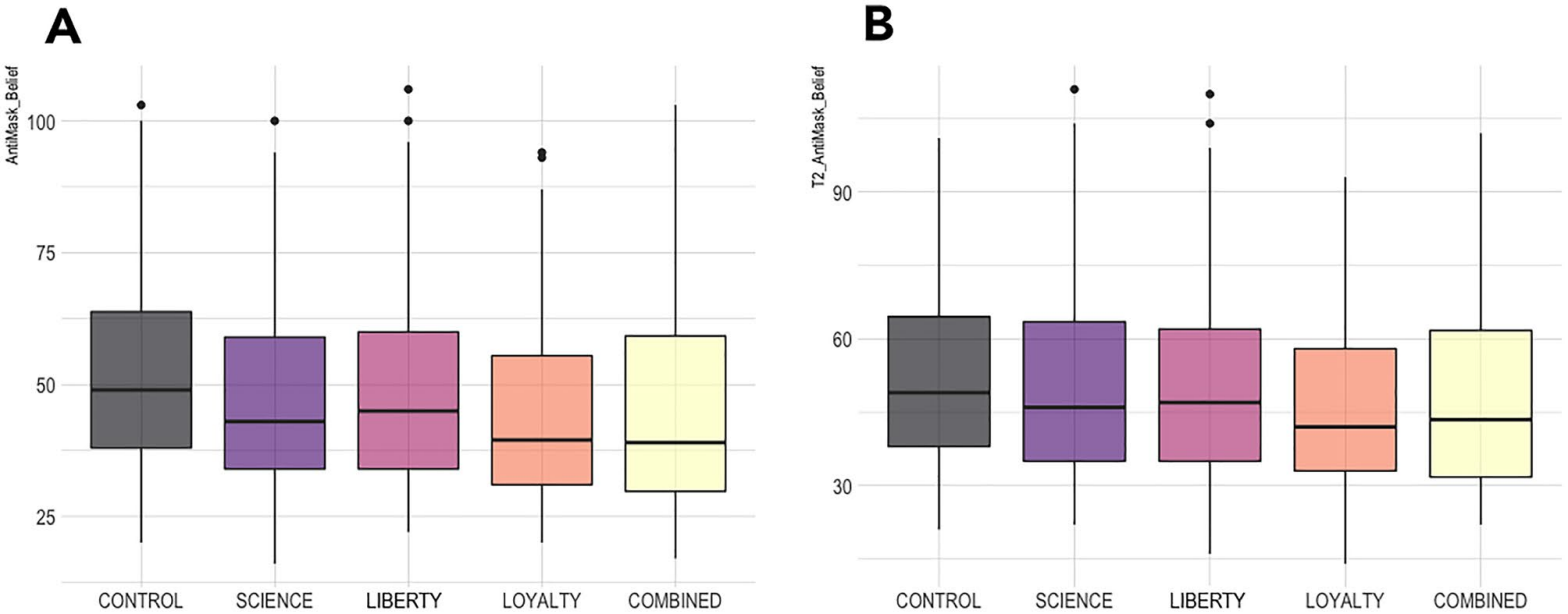
Study 2, anti-mask belief by video condition.

 The regression model for data at T1 significantly predicted Anti-mask Belief (F(8,588) = 2.846, p = 0.004), with R^2^ = 0.024 (*f* = 0.16), as did the variable of Conservatism (β = 2.564, t(588) = 2.493; 95% CI = 0.5439, 4.5832; p = 0.013; *f* = 0.01). The ANOVA test for a main effect of video type was significant (F(4,588) = 3.106, p = 0.016, partial η^2^ = 0.022). A full ANOVA table for this model is presented in Table S4. The Dunnett test comparing each group to CONTROL found significantly lower Anti-mask Belief for the group that saw the LOYALTY video (t(588) = − 3.083, p = 0.008) and for the group that saw the COMBINED video (t(588) = − 2.791, p = 0.02) (see Fig. 3A). The contrasts comparing CONTROL to LIBERTY (t(588) = − 1.01, p = 0.71) and to SCIENCE (t(588) = − 1.478, p = 0.389) were not significant. Full Dunnett statistics are presented in Table S5.

#### Test of H3

For data from T2, the overall regression model once again significantly predicted Anti-mask Belief (F(8,588) = 2.077, p = 0.036), with R^2^ = 0.0143 (*f* = 0.12), as did the variable of Conservatism (β = 2.577, t(588) = 2.499; 95% CI = 0.5517, 4.6036; p = 0.013; *f* = 0.01). The ANOVA model (Table S6) did not show a main effect of video type (F(8,588) = 1.87, p = 0.11), but the planned Dunnett comparisons (Table S7) showed that Anti-mask Belief was significantly lower in the group that had watched the LOYALTY video one week earlier compared with those who had seen the CONTROL video (t(588) = − 2.642, p = 0.03) (see Fig. 3B). The contrasts comparing COMBINED (t(588) = − 1.26, p = 0.54), LIBERTY (t(588) = − 0.716, p = 0.89), and SCIENCE (t(588) = − 0.869, p = 0.81) to CONTROL were not significant.

Reported mask wearing was generally high at T2 in crowded places (M = 87.56, SD = 28.0) and in public (M = 73.66, SD = 34.9), but lower around family and friends (M = 33.63, SD = 36.43). For mask wearing around family and friends at T2, the overall regression model did not predict mask wearing (F(8,575) = 1.33, p = 0.23), nor was there any effect of group in the ANOVA model (F(4,575) = 1.84, p = 0.12), nor any pairwise difference in the Dunnett test comparing each condition to CONTROL (COMBINED: t(588) = 0.95, p = 0.75; LIBERTY: t(588) = 1.94, p = 0.165; LOYALTY: t(588) = 2.23, p = 0.086; SCIENCE: t(588) = 2.112, p = 0.11). For mask wearing in crowded places at T2, the overall model was not significant (F(8,587) = 1.829, p = 0.069), nor was the main effect of Group (F(1,587) = 0.99, p = 0.41), nor any differences from CONTROL in the Dunnett test (COMBINED: t(588) = − 0.55, p = 0.95; LIBERTY: t(588) = − 1.31, p = 0.50; LOYALTY: t(588) = 0.283, p = 0.99; SCIENCE: t(588) = 0.279, p = 0.99), but Conservatism did negatively predict mask wearing ((β = − 4.04, t(588) = − 2.684; 95% CI = − 7.0045, − 1.0848; p = 0.008). For mask wearing in public, the overall model was not significant (F(2,588) = 0.464, p = 0.88), nor was there any effect of Group (F(4,587) = 0.273, p = 0.90), nor any differences from CONTROL in the Dunnett test (COMBINED: t(588) = 0.708, p = 0.89; LIBERTY: t(588) = 0.327, p = 0.99, LOYALTY: t(588) = 0.476, p = 0.97; SCIENCE: t(588) = 0.278, p = 0.99).

### Exploratory analysis

The first analysis modeled Anti-mask belief at T1 as a function of Group, Gender, Conservatism, Age, and Video Reaction. The full regression model significantly predicted Anti-Mask Belief (F(9,587) = 30.31, p < 0.001; R^2^ = 0.307; *f* = 0.67), as did the variables of Conservatism (β = 4.19, t(587) = 4.808; 95% CI = 2.4830, 5.9125; p < 0.001; *f* = 0.39) and Video Reaction (β = − 8.72, t(588) = − 15.516; 95% CI = − 9.8346, − 7.6246; p < 0.001; *f* = 0.41), showing that Conservatism was associated with higher anti-mask belief and Video Reaction was associated with lower anti-mask belief. The ANOVA showed a significant main effect of Group (F(4,587) = 4.37, p = 0.0172, partial η^2^ = 0.047), and the Dunnett test comparing each group to CONTROL showed significantly reduced Anti-Mask belief for those who saw the LOYALTY video (t(587) = − 4.71, p < 0.001) and the COMBINED video (t(587) = − 4.493, p < 0.001). In this analysis the LIBERTY and SCIENCE groups showed a trend towards reduced Anti-mask Belief that did not survive multiple comparisons correction (LIBERTY: t(587) = − 2.475, p = 0.047, SCIENCE: t(587) = − 2.537, p = 0.04).

The next analysis predicted Anti-mask belief at T2. The complete model predicted Anti-mask belief at T2 significantly, (F(9,587) = 27.35, p < 0.001, R^2^ = 0.28; *f* = 0.62), as did Conservatism (β = 4.17, t(587) = 4.71; 95% CI = 2.4360, 5.9118; p < 0.001; *f* = 0.04) and Video Reaction (β = − 8.523, t(588) = − 14.941; 95% CI = − 9.6434, − 7.4027; p < 0.001; *f* = 0.38). In the ANOVA model there was a trend towards a main effect of Group that did not survive multiple comparisons correction (F(4,587) = 2.58, p = 0.036, partial η^2^ = 0.029). The Dunnett test comparing each condition to CONTROL showed significantly reduced Anti-mask belief at T2 in participants who watched the LOYALTY video (t(587) = − 4.116, p < 0.001), and a trend towards reduced belief that did not survive multiple comparisons correction for those who watched the COMBINED video (t(587) = − 2.617, p = 0.032). The comparisons of LIBERTY (t(587) = − 2.068, p = 0.13) and SCIENCE (t(587) = − 1.77, p = 0.23) videos compared to control were not significant.

The model predicting frequency of mask wearing in public at T2 was significant, (F(9,586) = 10.88, p < 0.001; R^2^ = 0.13; *f* = 0.39) as was the variable of Video Reaction (β = 11.08, t(586) = 9.71; 95% CI = 8.84, 13.32; p < 0.001; *f* = 0.16). Conservatism did not survive multiple comparisons correction (β = − 3.99, t(587) = − 2.252; 95% CI = − 7.48, − 0.51; p = 0.025). There were no effects of Group overall or in the Dunnett test comparing each condition to CONTROL (COMBINED: t(587) = 1.53, p = 0.36; LIBERTY: t(587) = 1.18, p = 0.59; LOYALTY: t(587) = 1.19, p = 0.58; SCIENCE: t(587) = 0.217, p = 0.99).

The model predicting frequency of mask wearing in crowded places at T2 was significant, (F(9,586) = 6.01, p < 0.001; R^2^ = 0.07, *f* = 0.27) as were the variables of Video Reaction (β = 5.9, t(586) = 6.27; 95% CI = 4.05, 7.75; p < 0.001; *f* = 0.06) and Conservatism (β = − 5.14, t(586) =  − 3.518; 95% CI = − 8.01, − 2.27; p < 0.001; *f* = 0.02). There were no effects of Group overall (F(4,587) = 1.12, p = 0.34) or in the Dunnett test comparing each condition to CONTROL (COMBINED: t(587) = − 0.074, p = 0.99; LIBERTY: t(587) = − 0.834, p = 0.83; LOYALTY: t(587) = 0.72, p = 0.89; SCIENCE: t(587) = 0.60, p = 0.94).

The model predicting frequency of mask wearing around family and friends at T2 was significant, (F(9,584) = 15.09 p < 0.001, R^2^ = 0.18; *f* = 0.47) as was the variable of Video Reaction (β = 12.97, t(584) = 11.077; 95% CI = 10.67, 15.27; p < 0.001; *f* = 0.21). There was no main effect of Group in the ANOVA, (F(4,587) = 3.37, p = 0.09), but the Dunnett test showed significantly increased mask wearing for those who watched the LOYALTY video compared to the CONTROL video (t(587) 3.124, p = 0.007). Those who watched the LIBERTY video and the SCIENCE video showed a trend towards increased mask wearing that did not survive multiple comparisons correction (LIBERTY: t(587) = 3.031, p = 0.01, SCIENCE: t(587) = 2.865, p = 0.016). The COMBINED condition was not different from CONTROL (t(587) = 1.85, p = 0.199).

## Discussion

In our first study, we found that conservatives reported stronger anti-mask beliefs compared with moderates and liberals and were also less likely to report compliance with COVID safety protocols. Crucially, we found that these beliefs were related to underlying moral concerns. Anti-mask belief was positively associated with ingroup loyalty, and negatively associated with concern for harm and fairness. The association between anti-mask belief and with Identification with America suggests that national identity is specifically relevant to these beliefs. Furthermore, conservatives frequently reported not wearing masks due to concerns about freedom of choice and individual rights. These results are consistent with the idea that beliefs surrounding mask wearing in the United States have become moralized, and are thus linked to specific underlying moral concerns.

In our second, pre-registered study, we crafted messages about mask wearing framed to specifically target the moral concerns reported by conservatives. Consistent with other research^42,49,50,57,58^, we found that moral reframing of messages was successful. Video messages framed in terms of loyalty to America led to reduced anti-mask belief immediately following the video compared to a control video, and this reduction in anti-mask belief persisted for at least one week. In our exploratory analysis, when we included participants’ ratings of their reaction to the video in our model, we also found that those who watched the loyalty-framed video reported wearing their masks more frequently in the following week when they were around friends and family. The effect was not evident in other contexts (wearing masks in public or in crowded places), but those analyses are likely limited by a ceiling effect, since mask wearing was already very high in those contexts. When the information about masks was framed in terms of pure science, there was no significant reduction in anti-mask beliefs or change in mask-wearing behavior. This finding is consistent with research showing that scientific information is often rejected^30,59^, particularly when there is an underlying motivation to do so^60^.

The liberty framing did not have a significant effect on belief or behavior, despite indications from our data that this was an important value to our participants. This framing, which urged the wearing of masks in order to maintain individual liberty by avoiding government lockdowns, may have been unconvincing to those who did not believe strongly in the benefits of mask wearing to begin with. The concept that “Masks allow us to safely leave our homes, to interact with each other” seems to have been a bridge too far for our participants to accept. This result suggests that in order to be successful, moral reframing must not only resonate with the receiver’s moral concerns, but also must do so in a way that is sufficiently plausible to the audience. It is also possible that concern for liberty was not as strong as concern for loyalty among those who harbored anti-mask beliefs.

We hypothesized that the combined video, which contained elements of all three of the other frames (science, loyalty, liberty), would be more effective than the individual frames alone. The data did not support this hypothesis. The combined video did lead to decreased anti-mask belief immediately following the video, but this effect did not persist one week later, and was not accompanied by an increase in mask wearing. Given that elements of the loyalty frame appeared in the combined video, and the other frames did not lead to significant changes, it is reasonable to conclude that the limited effect the combined video had on belief was due to the presence of portions of the loyalty frame within the video. Furthermore, the measurement of anti-mask belief at the first time point, immediately following the video, may also be more susceptible to demand characteristics compared with the measurements one week later. Therefore, we consider the evidence that the combined video affected mask-wearing belief to be weak.

In exploratory analyses, we found that people’s ratings of the video as enjoyable, informative, compelling, and interesting, was a significant predictor of anti-mask belief following the videos immediately and one week later. This variable also predicted the frequency of mask wearing in all three contexts. It is possible that reaction to the videos captures some individual personality characteristic that might relate more directly to mask-related behavior. For example, video enjoyment has been found to correlate with openness to experience and agreeableness^61^. Since we do not have a measure of mask-related beliefs before the video, we cannot attribute this effect to the videos themselves. Nevertheless, the possibility that more engaging and interesting videos effect behavior more should be a topic of inquiry for future research. Evidence shows that more engaging messages that draw viewers in do tend to be more effective, which could be due to increased attention, or enhanced emotional responses to the stimuli^62,63^.

When we accounted for video reaction in our model, we found that watching the loyalty framed video, and that video alone, resulted in increased mask wearing during the week following viewing of the video. For health messages to be useful, they must ultimately translate to behavior aligned with the message, the relationships between beliefs about health and behavior is complex^64^. The success in increasing message-consistent behavior is therefore a promising result for moral reframing as a method of health messaging. Beliefs about mask-wearing alone with no accompanying behavior change would have no effect on the spread of a pandemic.

## Limitations

Given that our sample was not selected in a way to ensure representativeness across the entire population of conservatives in the United States, the generalizability of this result may be constrained. Nevertheless, we did not find any effects of gender, age, or any interaction between the effects of message type and any other variable in our experiment. This specific case of communicating about mask-wearing to American conservatives is complicated by the strong association between distrust in science and conservatism; a science framing is severely disadvantaged for this reason in this population. It is not necessarily always the case that information needs to be framed in terms of moral concerns in order to be accepted^28^.

The stimuli in our experiment were videos that contained a combination of text and moving images. We designed the stimuli this way to be as compelling and attention-worthy as possible, however, the disadvantage of this design is that we do not know whether the statements in the videos or the images, or some combination are responsible for the effects.

## Conclusions

These results provide further support that reframing messages to resonate with the values of an audience can improve the persuasiveness of those messages. In this specific case, framing scientific information about health-related behaviors in terms of the protection of one’s family, community, and nation, may be effective with conservative audiences that are otherwise resistant to such messaging.

## Supplementary Information

Supplementary Information.

## Data Availability

Raw data as well as analysis code in R are available on the Open Science Foundation project (https://osf.io/gmqfw/). All materials required to replicate the experiment are available online, including the videos which are on YouTube. Links to the stimuli are found in the Supplemental.
