# Supplementary material for: Moral reframing of messages about mask-wearing during the COVID-19 pandemic
Source: Sci Rep. 2023 Jun 22;13:10140. doi: 10.1038/s41598-023-37075-3 (PMC10287646; doi:10.1038/s41598-023-37075-3)
Supplement: Supplementary file 1 — Supplementary Information. [file 41598_2023_37075_MOESM1_ESM.docx]

**Supplemental Materials**

Survey Measures

*Moral Foundations Questionnaire***.** The short version of the Moral Foundations Questionnaire (MFQ20) is a 20 question self-report measure which assesses the degree to which individuals prioritize five moral domains in their decision-making (Graham et al., 2011). Subscales of the MFQ include: Harm/Care, Fairness/Reciprocity (alpha = 0.76), Ingroup/Loyalty (alpha = 0.68), Authority/Respect (alpha = 0.76), and Purity/Sanctity (alpha = 0.79). In Part 1 of the MFQ, respondents indicate (Graham et al., 2011) the relevance of various situational considerations to their decision-making about moral right or wrong using a six point scale from “not at all relevant” to “extremely relevant”. In Part 2 respondents indicate their agreement or disagreement with a series of moral statements, using a six-point scale from “strongly disagree” to “strongly agree”.

*Trust in Science and Scientists Inventory.* The Trust in Science and Scientists Inventory is a 21 question self-report measure which assesses an individual's domain-general level of trust in science and scientists (Nadelson et al., 2014). Questions assess trust in science in two areas: (1) perceptions of the nature of science, specifically the tentative nature of scientific knowledge, and (2) perceptions of certain research processes or scientist integrity. Respondents rank their level of agreement with a series of statements of trust in science and scientists in the two areas noted above, using a five-point scale from “strongly disagree” to “strongly agree”.  Internal consistency for this scale was high (alpha = 0.93).

*Identification With All Humanity (America/Community/All humanity).* The Identification With All Humanity (IWAH) is a nine-question self-report measure which assesses an individual's concern for the interests and concerns of all people (McFarland, Webb, & Brown, 2012). The IWAH has been shown to measure a construct which is not simply the absence of ethnocentrism and its correlates, and is more than the presence of empathy, general morality, universalism, and principled moral reasoning alone. Questions include “How often do you use the word ‘we’ to refer to the following groups of people?” and “How much would you say you have in common with the following groups?” Each of the 9 questions assess respondent identification with (1) “People in my community” (alpha = 0.85), (2) “Americans” (alpha = 0.83), and (4) “People all over the world” (alpha = 0.80). Respondents answer on a five-point scale from 1 (not at all) to 5 (very much).

*Very Short Authoritarianism Scale.* The Very Short Authoritarianism (VSA) Scale is a six-item self-report measure that is a shortened version of Altemeyer’s Right Wing Authoritarianism (RWA) scale (Bizumic & Duckitt, 2018). The RWA assesses three subdimensions of RWA: Authoritarian Submission, Authoritarian Aggression, and Conventionalism. The VSA was designed to assess the overall RWA construct, equally representing the three content subdimensions of the RWA while also balancing the direction of wording effects within these subdimensions as in the Authoritarianism Conservativism Traditionalism (ACT) scale (Duckitt & Bizumic, 2013). Respondents indicate agreement/disagreement with statements on a nine-point scale from “unsure/neutral” to “very strongly agree/disagree”. Internal consistency for this scale was high (alpha = 0.81).

*Anti-mask Belief (16 items).* The Anti-Mask Belief scale was constructed of 16 questions reflecting common sentiments expressed by individuals who advocate against mask-wearing, as well as beliefs they do not endorse which are shared by the scientific community and those for the use of masks. 14 of the questions were responded to on a 7-point scale from “Strongly Disagree” to “Strongly Agree”. The remaining two questions (“How effective do you believe wearing a mask is at protecting yourself from COVID-19?” and “How effective do you believe wearing a mask is at preventing you from transmitting COVID-19 to others?”) were responded to on a 7-point scale from “Not effective at all” to “Extremely effective”. Internal consistency for this measure was high (alpha = 0.87). The questions for this scale can be found in the below.

*COVID Guideline Adherence (12 items).* COVID Guideline Adherence consisted of 12 questions related to how often participants followed current health guidelines on preventing the spread of COVID-19 (social distancing, hand-washing, etc.). Questions were responded to on a 5 point scale from “Not at all” to “Always”. Internal consistency for this measure was high (alpha = 0.89). The questions for this scale can be found below.

*Mask-wearing reasons.* Participants were asked to click a check box next to any of several reasons why they might not wear a mask. Those reasons are listed below.

**Video stimuli descriptions.**

CONTROL: A short video about trains, which contained no information about mask-wearing.

SCIENCE: Scientific information about the benefits of mask-wearing

LIBERTY: Moral reframing of mask-wearing based on the value of liberty. The main message of the video was that because wearing masks keeps the disease from spreading, it prevents government interference in the form of lockdowns, and allows people the freedom associated with restarting the economy and returning to work.

LOYALTY: Provided moral reframing of mask-wearing based on the value of loyalty to the community and to America. The main message of the video was that because wearing masks keeps the disease from spreading, it is a way of caring for our community and protecting America of the threat of the disease.

COMBINED: This video combined elements from the SCIENCE, LIBERTY, and LOYALTY videos. It presented some scientific information about the benefits of mask-wearing, as well as moral reframing of mask-wearing in terms of liberty and loyalty. The text of the videos is in the appendix, as are the links to the videos on YouTube.

**Stimulus Validation.**

Six self-described experts in the MFT watched each of the five videos, in random order, and rated for each video how strongly they felt one of six values were represented in the video (Care/Harm, Fairness/Cheating, Loyalty/Betrayal, Authority/Subversion, Sanctity/Degradation, Liberty/Oppression) on a scale from 0-10. The results are show in Figure S1. The raters found little to no moral content in the CONTROL video, as expected. In the SCIENCE video, the most highly rated value was Harm/Care. In the LIBERTY video the most commonly rated value was Liberty/Oppression, as expected. In the LOYALTY video the most commonly rated value was Loyalty as expected, but raters also appeared to identify substantial reference to Liberty/Oppression in this video as well. The COMBINED video reflected multiple values according to the raters, including substantial Harm/Care, Loyalty/Betrayal, and Liberty/Oppression.

**Figure S1: Expert ratings of the moral content of the video stimuli**


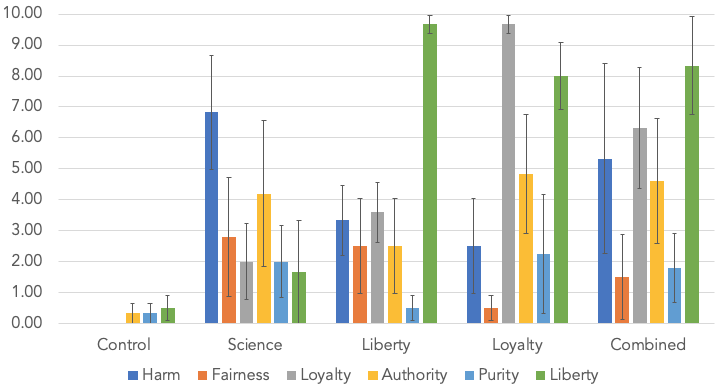


**Exclusion Criteria**

Study 1: We excluded any subjects who had more than one item unanswered in any of the measures.

Study 2: The exlusion criteria for this study were pre-registered as:

• Following each video there will be two questions about the content. If a participant answers both questions incorrectly, they will be excluded from analysis.

• Additionally, since our target population are self-identified conservatives, participants will be excluded if they answer that they are not conservative on our demographic questionnaire (a score of less than 5 on a 7 point scale where 1 is very liberal, and 7 is very conservative).

• Additionally, since it is important that each person has watched the stimulus video, if the timing of the survey indicates that they spend less than 2 minutes on the video slide, they will be excluded.

**Study 1: Correlation results**

There were significant relationships among many of the measured variables in Study 1, depicted in Figure S2. Notably, Anti-mask Belief correlated negatively with Trust in Science (r(531) = -0.633, p < 0.0001), COVID Guideline Adherence (r(531) = -0.583, p < .0001), MFQ Harm/Care (r(531) = -0.365, p < 0.0001), MFQ Fairness/Reciprocity (r(531) = -0.361, p < 0.0001), and positively with MFQ Purity/Sanctity (r(531) = 0.252, p < 0.0001, MFQ Ingroup/Loyalty (r(531)=0.356, p < .0001), MFQ Authority/Respect (r(531) = 0.354, p < 0.0001), Authoritarianism (r(531) = 0.426, p < 0.0001), Conservatism (r(531) = 0.197, p = 0.0007), and Identification with America (r(531) = 0.177, p < 0.0001) scores. COVID Guideline Adherence correlated positively with MFQ Harm/Care (r(531) = 0.390, p < 0.0001), MFQ Fairness/Reciprocity (r(531) = 0.372, p < 0.0001), Identification with Community (r(531) = 0.248, p < 0.0001), and Trust in Science (r(531) = 0.313, p < 0.0001), and negatively with Anti-mask Belief (r(531) = -0.583, p < 0.0001) and Authoritarianism (r(531) = -0.214, p < 0.0001).

**Table S1: Correlations in Study 1**

|  | **Cons** | **TIS** | **VSA** | **Harm** | **Fairness** | **Loyalty** | **Authority** | **Purity** | **IWA** | **IWC** | **AB** | **CGA** | **IWAH** |
| --- | --- | --- | --- | --- | --- | --- | --- | --- | --- | --- | --- | --- | --- |
| **Cons** | 1 |  |  |  |  |  |  |  |  |  |  |  |  |
| **TIS** | -0.183* | 1 |  |  |  |  |  |  |  |  |  |  |  |
| **VSA** | 0.260*** | -0.515*** | 1 |  |  |  |  |  |  |  |  |  |  |
| **Harm** | -0.098 | 0.253*** | -0.191* | 1 |  |  |  |  |  |  |  |  |  |
| **Fairness** | -0.08 | 0.297*** | -0.165 | 0.652*** | 1 |  |  |  |  |  |  |  |  |
| **Loyalty** | 0.246*** | -0.369*** | 0.578*** | 0.056 | 0.05 | 1 |  |  |  |  |  |  |  |
| **Authority** | 0.281*** | -0.445*** | 0.701*** | 0.049 | 0.042 | 0.772*** | 1 |  |  |  |  |  |  |
| **Purity** | 0.213** | -0.379*** | 0.549*** | 0.154 | 0.096 | 0.568*** | 0.701*** | 1 |  |  |  |  |  |
| **IWA** | 0.192* | -0.154 | 0.360*** | 0.173*** | 0.157 | 0.512*** | 0.424*** | 0.348*** | 1 |  |  |  |  |
| **IWC** | 0.033 | -0.005 | 0.131 | 0.324*** | 0.246* | 0.329*** | 0.259*** | 0.219*** | 0.625*** | 1 |  |  |  |
| **AB** | 0.197** | -0.633*** | 0.426*** | -0.365*** | -0.361*** | 0.356*** | 0.354*** | 0.252*** | 0.177* | -0.027 | 1 |  |  |
| **CGA** | -0.065 | 0.313*** | -0.214*** | 0.391*** | 0.372*** | -0.092 | -0.08 | -0.018 | 0.045 | 0.248*** | -0.583*** | 1 |  |
| **IWAH** | -0.011 | 0.091 | -0.087 | 0.385*** | 0.288*** | 0.154 | 0.065 | 0.141 | 0.543*** | 0.496*** | -0.144 | 0.207** | 1 |

*Cons = conservativism, TIS = Trust in Science, VSA = Very Short Authoritarianism, Harm = MFQ Harm, Fairness = MFQ Fairness, Loyalty = MFQ Loyalty, Purity = MFQ Purity, IWA = Identification With America, IWC = Identification with Community, AB = Anti-mask Belief, CGA = COVID Guideline Adherence, IWAH = Identification With All Humanity*

*Corrected p values: *p < 0.01, **p< 0.001, ***p<0.0001*

**Figure S2: Correlations among measurements in Study 1**


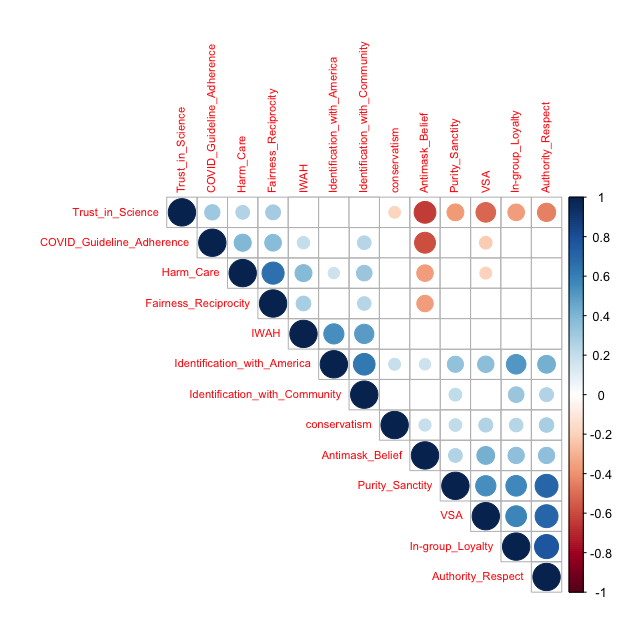


**Table S2: Study 1 regression table**

|  | **Antimask Belief** | | |
| --- | --- | --- | --- |
| *Predictors* | *Estimates* | *CI* | *p* |
| (Intercept) | 98.35 | 89.39 – 107.31 | **<0.001** |
| conservatism | 0.16 | -0.26 – 0.57 | 0.456 |
| Trust in Science | -0.53 | -0.62 – -0.45 | **<0.001** |
| VSA | -0.02 | -0.16 – 0.12 | 0.767 |
| Harm Care | -0.68 | -1.06 – -0.30 | **0.001** |
| Fairness Reciprocity | -0.50 | -0.88 – -0.13 | **0.009** |
| In-group Loyalty | 0.55 | 0.19 – 0.92 | **0.003** |
| Authority Respect | -0.02 | -0.45 – 0.40 | 0.920 |
| Purity Sanctity | -0.02 | -0.31 – 0.27 | 0.893 |
| Identification with America | 0.33 | 0.12 – 0.55 | **0.002** |
| Identification with Community | -0.10 | -0.28 – 0.07 | 0.242 |
| IWAH | -0.20 | -0.39 – -0.02 | **0.031** |
| Observations | 533 | | |
| R^2^ / R^2^ adjusted | 0.493 / 0.482 | | |

**Table S3: Tolerance and Variance Inflation Factors**

**Study 1**

|  | Variables | Tolerance | VIF |
| --- | --- | --- | --- |
| 1 | conservatism | 0.8883776 | 1.125648 |
| 2 | Trust_in_Science | 0.6434149 | 1.554207 |
| 3 | VSA | 0.3909921 | 2.557596 |
| 4 | Harm_Care | 0.4886864 | 2.046302 |
| 5 | Fairness_Reciprocity | 0.544751 | 1.835701 |
| 6 | Ingroup_Loyalty | 0.3592111 | 2.783878 |
| 7 | Authority_Respect | 0.2470145 | 4.048346 |
| 8 | Purity_Sanctity | 0.4649383 | 2.150823 |
| 9 | Identification_with_America | 0.3889927 | 2.570742 |
| 10 | Identification_with_Community | 0.5405519 | 1.849961 |
| 11 | IWAH | 0.5459371 | 1.831713 |

**Study 2**

|  | | Variables | Tolerance | VIF |
| --- | --- | --- | --- | --- |
| 1 | GroupCONTROL | | 0.6182996 | 1.617339 |
| 2 | GroupLIBERTY | | 0.6220512 | 1.607585 |
| 3 | GroupLOYALTY | | 0.6451586 | 1.550006 |
| 4 | GroupSCIENCE | | 0.6487221 | 1.541492 |
| 5 | GenderMale | | 0.9686494 | 1.032365 |
| 7 | | Conservatism | 0.9797188 | 1.020701 |
| 8 | | Age | 0.956801 | 1.045149 |

**Table S4: Study 2 Regression Table for T1**

|  | **AntiMask Belief** | | |
| --- | --- | --- | --- |
| *Predictors* | *Estimates* | *CI* | *p* |
| (Intercept) | 35.19 | 22.52 – 47.85 | **<0.001** |
| Group [COMBINED] | -6.69 | -11.40 – -1.98 | **0.005** |
| Group [LIBERTY] | -2.45 | -7.21 – 2.30 | 0.312 |
| Group [LOYALTY] | -7.57 | -12.39 – -2.75 | **0.002** |
| Group [SCIENCE] | -3.65 | -8.50 – 1.20 | 0.140 |
| Group [contrast] | -0.53 | -1.51 – 0.45 | 0.286 |
| Gender | -1.53 | -4.66 – 1.60 | 0.337 |
| Conservatism | 2.56 | 0.54 – 4.58 | **0.013** |
| Age | 0.07 | -0.04 – 0.18 | 0.206 |
| Observations | 597 | | |
| R^2^ / R^2^ adjusted | 0.037 / 0.024 | | |

**Table S4: Study 2 ANOVA Table Anti-mask Belief at T1**

|  | DF | MS | F | P-value |  |
| --- | --- | --- | --- | --- | --- |
| Group | 4 | 1122.05 | 3.1062 | **0.01515** | ***** |
| Gender | 2 | 344.05 | 0.9525 | 0.38639 |  |
| Conservatism | 1 | 2468.59 | 6.8339 | **0.00917** | ****** |
| Age | 1 | 579.66 | 1.6047 | 0.20574 |  |
| Residuals | 588 | 361.23 |  |  |  |

**Table S5: Dunnett Contrasts for Anti-mask Belief at T1**

|  | Estimate | Std. Error | t value | P-value |  |
| --- | --- | --- | --- | --- | --- |
| Combined-Control | -6.691 | 2.397 | -2.791 | **0.01968** | ***** |
| Liberty-Control | -2.453 | 2.422 | -1.013 | 0.71016 |  |
| Loyalty-Control | -7.571 | 2.455 | -3.083 | **0.00813** | ****** |
| Science-Control | -3.649 | 2.468 | -1.478 | 0.3888 |  |

**Table S6: Study 2 Regression Tables for T2**

|  | **T2_AntiMask_Belief** | | |
| --- | --- | --- | --- |
| *Predictors* | *Estimates* | *CI* | *p* |
| (Intercept) | 32.28 | 19.33 – 45.23 | **<0.001** |
| Group [CONTROL] | 3.02 | -1.70 – 7.74 | 0.210 |
| Group [LIBERTY] | 1.28 | -3.55 – 6.11 | 0.603 |
| Group [LOYALTY] | -3.49 | -8.37 – 1.40 | 0.162 |
| Group [SCIENCE] | 0.87 | -4.04 – 5.78 | 0.728 |
| Group [contrast] | 0.11 | -0.87 – 1.10 | 0.824 |
| Age | 0.06 | -0.05 – 0.17 | 0.272 |
| Gender | -0.63 | -3.77 – 2.51 | 0.694 |
| Conservatism | 2.58 | 0.55 – 4.60 | **0.013** |
| Observations | 597 | | |
| R^2^ / R^2^ adjusted | 0.027 / 0.014 | | |
| \|  \| **T2_MaskWearing_FamilyFriends** \| \| \| \| --- \| --- \| --- \| --- \| \| *Predictors* \| *Estimates* \| *CI* \| *p* \| \| (Intercept) \| 26.53 \| 1.98 – 51.08 \| **0.034** \| \| Group [COMBINED] \| 4.41 \| -4.68 – 13.50 \| 0.341 \| \| Group [LIBERTY] \| 9.09 \| -0.08 – 18.26 \| 0.052 \| \| Group [LOYALTY] \| 10.63 \| 1.30 – 19.95 \| **0.026** \| \| Group [SCIENCE] \| 10.10 \| 0.71 – 19.50 \| **0.035** \| \| Age \| -0.17 \| -0.39 – 0.04 \| 0.116 \| \| Gender \| -2.50 \| -8.57 – 3.56 \| 0.418 \| \| Conservatism \| 1.44 \| -2.46 – 5.35 \| 0.468 \| \| Observations \| 584 \| \| \| \| R^2^ / R^2^ adjusted \| 0.018 / 0.005 \| \| \|  \|  \| **T2_MaskWearing_CrowdedPlaces** \| \| \| \| --- \| --- \| --- \| --- \| \| *Predictors* \| *Estimates* \| *CI* \| *p* \| \| (Intercept) \| 110.66 \| 92.22 – 129.10 \| **<0.001** \| \| Group [COMBINED] \| -1.91 \| -8.78 – 4.95 \| 0.584 \| \| Group [LIBERTY] \| -4.63 \| -11.55 – 2.30 \| 0.190 \| \| Group [LOYALTY] \| 1.01 \| -6.01 – 8.03 \| 0.777 \| \| Group [SCIENCE] \| 1.00 \| -6.05 – 8.06 \| 0.780 \| \| Age \| -0.01 \| -0.17 – 0.16 \| 0.949 \| \| Gender \| 2.78 \| -1.77 – 7.34 \| 0.231 \| \| Conservatism \| -4.04 \| -6.98 – -1.10 \| **0.007** \| \| Observations \| 596 \| \| \| \| R^2^ / R^2^ adjusted \| 0.023 / 0.010 \| \| \|   **T2_MaskWearing_Public** | | |  |
| *Predictors* | *Estimates* | *CI* | *p* |
| (Intercept) | 85.42 | 62.11 – 108.73 | **<0.001** |
| Group [COMBINED] | 3.13 | -5.55 – 11.80 | 0.479 |
| Group [LIBERTY] | 1.46 | -7.31 – 10.23 | 0.744 |
| Group [LOYALTY] | 2.16 | -6.73 – 11.04 | 0.634 |
| Group [SCIENCE] | -1.26 | -10.20 – 7.67 | 0.781 |
| Age | -0.07 | -0.27 – 0.14 | 0.519 |
| Gender | 1.20 | -4.56 – 6.96 | 0.683 |
| Conservatism | -1.87 | -5.60 – 1.85 | 0.323 |
| Observations | 596 | | |
| R^2^ / R^2^ adjusted | 0.005 / -0.008 | | |

**Table S6: Study 2 ANOVA Tables for T2**

**Anti-mask Belief**

|  | DF | MS | F | P-value |  |
| --- | --- | --- | --- | --- | --- |
| Group | 4 | 681.2 | 1.874 | 0.1134 |  |
| Age | 1 | 684.2 | 1.882 | 0.1706 |  |
| Gender | 2 | 180.4 | 0.496 | 0.609 |  |
| Conservatism | 1 | 2269.8 | 6.244 | **0.0127** | ***** |
| Residuals | 588 | 363.5 |  |  |  |

**Mask-wearing Behavior – Around Friends & Family**

|  | DF | MS | F | P-value |
| --- | --- | --- | --- | --- |
| Group | 4 | 2440.3 | 1.841 | 0.119 |
| Age | 1 | 2508.7 | 1.892 | 0.169 |
| Gender | 2 | 697.3 | 0.526 | 0.591 |
| Conservatism | 1 | 698.9 | 0.527 | 0.468 |
| Residuals | 575 | 1325.6 |  |  |

**Mask-wearing Behavior – In Crowded Places**

|  | DF | MS | F | P-value |
| --- | --- | --- | --- | --- |
| Group | 4 | 763 | 0.998 | 0.40817 |
| Age | 1 | 160 | 0.209 | 0.64769 |
| Gender | 2 | 939 | 1.226 | 0.2941 |
| Conservatism | 1 | 5564 | 7.27 | **0.00721**** |
| Residuals | 587 | 765 |  |  |

**Mask-wearing Behavior – In Public**

|  | DF | MS | F | P-value |
| --- | --- | --- | --- | --- |
| Group | 4 | 333.8 | 0.273 | 0.895 |
| Age | 1 | 751.3 | 0.615 | 0.433 |
| Gender | 2 | 288.6 | 0.236 | 0.79 |
| Conservatism | 1 | 1194.7 | 0.978 | 0.323 |
| Residuals | 587 | 1221.9 |  |  |

**Table S7: Dunnett Contrasts T2**

**Anti-mask belief**

|  | Estimate | Std. Error | t value | P-value |  |
| --- | --- | --- | --- | --- | --- |
| Combined-Control | -3.02 | 2.404 | -1.256 | 0.5367 |  |
| Liberty -Control | -1.74 | 2.429 | -0.716 | 0.8897 |  |
| Loyalty-Control | -6.507 | 2.463 | -2.642 | **0.0301** | ***** |
| Science-Control | -2.151 | 2.476 | -0.869 | 0.8056 |  |

**Mask-wearing Behavior – Around Friends & Family**

|  | Estimate | Std. Error | t value | P-value |
| --- | --- | --- | --- | --- |
| Combined-Control | 4.412 | 4.629 | 0.953 | 0.752 |
| Liberty -Control | 9.091 | 4.67 | 1.947 | 0.165 |
| Loyalty-Control | 10.626 | 4.749 | 2.238 | 0.086 |
| Science-Control | 10.103 | 4.784 | 2.112 | 0.115 |

**Mask-wearing Behavior – In Crowded Places**

|  | Estimate | Std. Error | t value | P-value |
| --- | --- | --- | --- | --- |
| Combined-Control | -1.915 | 3.494 | -0.548 | 0.954 |
| Liberty -Control | -4.627 | 3.525 | -1.313 | 0.498 |
| Loyalty-Control | 1.012 | 3.574 | 0.283 | 0.996 |
| Science-Control | 1.002 | 3.593 | 0.279 | 0.996 |

**Mask-wearing Behavior – In Public**

|  | Estimate | Std. Error | t value | P-value |
| --- | --- | --- | --- | --- |
| Combined-Control | 3.126 | 4.417 | 0.708 | 0.893 |
| Liberty-Control | 1.46 | 4.463 | 0.327 | 0.993 |
| Loyalty-Control | 2.156 | 4.524 | 0.476 | 0.972 |
| Science-Control | -1.263 | 4.548 | -0.278 | 0.996 |

**Table S8: Exploratory Analyses**

**Regression tables**

|  | **AntiMask Belief at T1** | | |
| --- | --- | --- | --- |
| *Predictors* | *Estimates* | *CI* | *p* |
| (Intercept) | 55.47 | 44.50 – 66.45 | **<0.001** |
| Group [COMBINED] | -9.10 | -13.08 – -5.12 | **<0.001** |
| Group [LIBERTY] | -5.07 | -9.09 – -1.05 | **0.014** |
| Group [LOYALTY] | -9.77 | -13.85 – -5.70 | **<0.001** |
| Group [SCIENCE] | -5.28 | -9.38 – -1.19 | **0.011** |
| Gender | -0.23 | -2.87 – 2.42 | 0.867 |
| Conservatism | 4.20 | 2.48 – 5.91 | **<0.001** |
| Age | 0.08 | -0.01 – 0.17 | 0.094 |
| Video Reaction | -8.73 | -9.83 – -7.62 | **<0.001** |
| Observations | 597 | | |
| R^2^ / R^2^ adjusted | 0.317 / 0.307 | | |

|  | **AntiMask Belief at T2** | | |
| --- | --- | --- | --- |
| *Predictors* | *Estimates* | *CI* | *p* |
| (Intercept) | 55.10 | 43.97 – 66.23 | **<0.001** |
| Group [COMBINED] | -5.38 | -9.41 – -1.34 | **0.009** |
| Group [LIBERTY] | -4.29 | -8.37 – -0.22 | **0.039** |
| Group [LOYALTY] | -8.66 | -12.79 – -4.53 | **<0.001** |
| Group [SCIENCE] | -3.75 | -7.90 – 0.40 | 0.077 |
| Gender | 0.65 | -2.03 – 3.33 | 0.636 |
| Conservatism | 4.17 | 2.43 – 5.91 | **<0.001** |
| Age | 0.07 | -0.02 – 0.16 | 0.145 |
| Video Reaction | -8.52 | -9.64 – -7.40 | **<0.001** |
| Observations | 597 |  |  |
| R^2^ / R^2^ adjusted | 0.295 / 0.285 | | |
| \|  \| **T2_MaskWearing_Public** \| \| \| \| --- \| --- \| --- \| --- \| \| *Predictors* \| *Estimates* \| *CI* \| *p* \| \| (Intercept) \| 66.11 \| 43.56 – 88.67 \| **<0.001** \| \| Group [CONTROL] \| -6.30 \| -14.38 – 1.79 \| 0.127 \| \| Group [LIBERTY] \| -1.41 \| -9.64 – 6.82 \| 0.737 \| \| Group [LOYALTY] \| -1.24 \| -9.57 – 7.08 \| 0.769 \| \| Group [SCIENCE] \| -5.38 \| -13.74 – 2.98 \| 0.207 \| \| Age \| -0.08 \| -0.27 – 0.11 \| 0.419 \| \| Gender \| -0.41 \| -5.78 – 4.95 \| 0.879 \| \| Conservatism \| -3.99 \| -7.48 – -0.51 \| **0.025** \| \| Video Reaction \| 11.08 \| 8.84 – 13.32 \| **<0.001** \| \| Observations \| 596 \|  \|  \| \| R^2^ / R^2^ adjusted \| 0.143 / 0.130 \| \| \| \|  \|  \| \| \|  \|  \| **T2_MaskWearing_CrowdedPlaces** \| \| \| \| --- \| --- \| --- \| --- \| \| *Predictors* \| *Estimates* \| *CI* \| *p* \| \| (Intercept) \| 96.66 \| 78.07 – 115.26 \| **<0.001** \| \| Group [CONTROL] \| 0.25 \| -6.42 – 6.92 \| 0.941 \| \| Group [LIBERTY] \| -2.61 \| -9.41 – 4.20 \| 0.452 \| \| Group [LOYALTY] \| 2.75 \| -4.13 – 9.63 \| 0.433 \| \| Group [SCIENCE] \| 2.36 \| -4.56 – 9.27 \| 0.504 \| \| Age \| -0.01 \| -0.17 – 0.15 \| 0.900 \| \| Gender \| 1.89 \| -2.54 – 6.31 \| 0.402 \| \| Conservatism \| -5.14 \| -8.01 – -2.27 \| **<0.001** \| \| Video Reaction \| 5.90 \| 4.05 – 7.75 \| **<0.001** \| \| Observations \| 596 \|  \|  \| \| R^2^ / R^2^ adjusted \| 0.085 / 0.070 \| \| \| \|  \|  \| \| \|  \|  \| **T2_MaskWearing_FamilyFriends** \| \| \| \| --- \| --- \| --- \| --- \| \| *Predictors* \| *Estimates* \| *CI* \| *p* \| \| (Intercept) \| 2.77 \| -20.48 – 26.02 \| 0.815 \| \| Group [CONTROL] \| -7.81 \| -16.10 – 0.47 \| 0.064 \| \| Group [FREEDOM] \| 5.09 \| -3.40 – 13.57 \| 0.239 \| \| Group [LOYALTY] \| 5.69 \| -2.90 – 14.28 \| 0.194 \| \| Group [SCIENCE] \| 4.65 \| -4.00 – 13.31 \| 0.292 \| \| Age \| -0.17 \| -0.36 – 0.03 \| 0.096 \| \| Gender \| -4.48 \| -10.00 – 1.04 \| 0.112 \| \| Conservatism \| -0.86 \| -4.43 – 2.71 \| 0.636 \| \| Video Reaction \| 12.97 \| 10.67 – 15.27 \| **<0.001** \| \| Observations \| 584 \|  \|  \| \| R^2^ / R^2^ adjusted \| 0.191 / 0.179 \| \| \| \|  \|  \| \| \| |  | | |

**ANOVA Tables**

| **Anti-mask Belief at T1** | DF | MS | F | P-value |  |
| --- | --- | --- | --- | --- | --- |
| Group | 4 | 1122 | 4.373 | **0.00172** | ** |
| Gender | 2 | 344 | 1.341 | 0.26243 |  |
| Conservatism | 1 | 2469 | 9.62 | **0.00202** | ** |
| Age | 1 | 580 | 2.259 | 0.13338 |  |
| Video Reaction | 1 | 61776 | 240.746 | **< 2E-16** | *** |
| Residuals | 587 | 257 |  |  |  |

**Anti-mask Belief at T2**

|  | DF | MS | F | P-value |  |
| --- | --- | --- | --- | --- | --- |
| Group | 4 | 681 | 2.582 | 0.03632 |  |
| Gender | 2 | 204 | 0.775 | 0.46138 |  |
| Conservatism | 1 | 2467 | 9.353 | **0.00233** | ** |
| Age | 1 | 439 | 1.663 | 0.19764 |  |
| Video Reaction | 1 | 58887 | 223.23 | **< 2E-16** | *** |
| Residuals | 587 | 264 |  |  |  |

**Mask-wearing In Public**

|  | DF | MS | F | P-value |  |
| --- | --- | --- | --- | --- | --- |
| Group | 4 | 334 | 0.317 | 0.867 |  |
| Age | 1 | 751 | 0.713 | 0.399 |  |
| Gender | 2 | 289 | 0.274 | 0.761 |  |
| Conservatism | 1 | 1195 | 1.133 | 0.288 |  |
| Video Reaction | 1 | 99395 | 94.27 | **<2e-16** | *** |
| Residuals | 586 | 1054 |  |  |  |

**Mask-wearing in Crowded Places**

|  | DF | MS | F | P-value |  |
| --- | --- | --- | --- | --- | --- |
| Group | 4 | 763 | 1.063 | 0.37415 |  |
| Age | 1 | 160 | 0.223 | 0.63719 |  |
| Gender | 2 | 939 | 1.306 | 0.2716 |  |
| Conservatism | 1 | 5564 | 7.744 | **0.00556** | ** |
| Video Reaction | 1 | 28208 | 39.263 | **7.18E-10** | *** |
| Residuals | 586 | 718 |  |  |  |

**Mask-wearing around Friends & Family**

|  | DF | MS | F | P-value |  |
| --- | --- | --- | --- | --- | --- |
| Group | 4 | 2440 | 2.231 | 0.0645 | . |
| Age | 1 | 2509 | 2.293 | 0.1305 |  |
| Gender | 2 | 697 | 0.637 | 0.5291 |  |
| Conservatism | 1 | 699 | 0.639 | 0.4245 |  |
| Video Reaction | 1 | 134252 | 122.709 | **<2e-16** | *** |
| Residuals | 574 | 1094 |  |  |  |

**Dunnett Tests**

**Anti-mask Belief at T1**

|  | Estimate | Std. Error | t value | P-value |  |
| --- | --- | --- | --- | --- | --- |
| Combined-Control | -9.104 | 2.026 | -4.493 | **<0.001** | ******* |
| Liberty-Control | -5.069 | 2.048 | -2.475 | 0.0473 |  |
| Loyalty-Control | -9.773 | 2.074 | -4.711 | **<0.001** | ******* |
| Science-Control | -5.285 | 2.083 | -2.537 | 0.04 |  |

**Anti-mask Belief at T2**

|  | Estimate | Std. Error | t value | P-value |  |
| --- | --- | --- | --- | --- | --- |
| Combined-Control | -5.376 | 2.054 | -2.617 | 0.0324 |  |
| Liberty -Control | -4.294 | 2.077 | -2.068 | 0.1266 |  |
| Loyalty-Control | -8.657 | 2.103 | -4.116 | **<0.001** | ******* |
| Science-Control | -3.747 | 2.112 | -1.774 | 0.2316 |  |

**Mask wearing in Public**

|  | Estimate | Std. Error | t value | P-value |
| --- | --- | --- | --- | --- |
| Combined-Control | 6.2965 | 4.1165 | 1.53 | 0.357 |
| Liberty-Control | 4.8882 | 4.1612 | 1.175 | 0.593 |
| Loyalty-Control | 5.052 | 4.213 | 1.199 | 0.576 |
| Science-Control | 0.9177 | 4.231 | 0.217 | 0.999 |

**Mask wearing in Crowded Places**

|  | Estimate | Std. Error | t value | P-value |
| --- | --- | --- | --- | --- |
| Combined-Control | -0.2505 | 3.3962 | -0.074 | 1 |
| Liberty-Control | -2.8583 | 3.4271 | -0.834 | 0.826 |
| Loyalty-Control | 2.4981 | 3.471 | 0.72 | 0.888 |
| Science-Control | 2.1055 | 3.4856 | 0.604 | 0.936 |

**Mask wearing around Family & Friends**

|  | Estimate | Std. Error | t value | P-value |  |
| --- | --- | --- | --- | --- | --- |
| Combined-Control | 7.815 | 4.217 | 1.853 | 0.19921 |  |
| Liberty-Control | 12.901 | 4.256 | 3.031 | 0.00958 |  |
| Loyalty-Control | 13.503 | 4.322 | 3.124 | **0.00708** | ****** |
| Science-Control | 12.467 | 4.351 | 2.865 | 0.01588 |  |

**Survey Measures**

**Anti-mask Belief (16 items)**

For the following statements, indicate how strongly you agree or disagree
(7 point scale: “Strongly disagree”, “Disagree”, “Moderately disagree”, “Neither agree nor Disagree”, “Slightly agree”, “Moderately agree”, “Strongly agree”)

1. Wearing a mask is a sign of weakness
2. Wearing a mask is a sign of being a follower
3. (-) Wearing a mask is a sign of caring for others
4. (-) Science shows that wearing masks is effective to prevent disease transmission
5. Government-mandated mask wearing is an attack on individual freedom
6. Wearing a mask is uncomfortable
7. My decision to wear a mask predominantly affects me
8. (-) My decision to wear a mask predominantly affects others
9. (-) Businesses should have a right to refuse entry and service to people NOT wearing masks
10. Businesses should have a right to refuse entry and services to people who ARE wearing masks
11. I look down on people who wear masks in public
12. (-) I look down on people who do not wear masks in public
13. Masks are helpful for doctors and healthcare professionals, but not for me
14. People look silly when wearing masks in public

15. (-) How effective do you believe wearing a mask is in protecting yourself from COVID-19? (scored on a 1-7 scale labeled “not effective at all”, ”somewhat effective”, “extremely effective” at the 1, 4, 7 marks respectively)

16. (-) How effective do you believe wearing a mask is in preventing you from transmitting COVID-19 to others? (scored on a 1-7 scale labeled “not effective at all”, ”somewhat effective”, “extremely effective” at the 1, 4, 7 marks respectively)

**COVID Guideline Adherence (12 items)**

To what extent do you act in accordance with the following COVID-19 prevention guidelines? (5 point scale: “Not at all”, “Sometimes”, “About half the time”, “Most of the time”, “Always”)

1. Regularly and thoroughly cleaning your hands with an alcohol-based hand rub
2. Avoiding touching your eyes, nose and mouth with unwashed hands
3. Covering your mouth and nose with your bent elbow or tissue when you cough or sneeze
4. Frequently washing your hands with soap and water for at least 20 seconds
5. Avoiding meetings, events, and other social gatherings
6. Practicing social distancing by doing your grocery shopping at off-peak hours and/or less often
7. Maintaining at least 6 feet distance between yourself and others
8. Practicing social distancing by avoiding crowds in confined and poorly ventilated spaces
9. Avoiding contact with sick people
10. Regularly cleaning and disinfecting frequently touched surfaces
11. Staying home if you are sick, or, hypothetically staying home if you were sick (except to get medical care).
12. Wearing a mask when you leave the house.

**Mask-Wearing Reasons.** Participants were also asked to click a check box next to any of several reasons why they might not wear a mask. Those reasons included:

Because masks are uncomfortable

Because I don't think it will prevent me from getting sick

Because I don't think it will prevent others from getting sick

Because it looks silly

Because it's my choice not to wear one

Because it looks weak

Because I don't have one

Because it fogs up my glasses

Because my friends aren't wearing them

Because it is socially awkward to wear one

Because it is my right not to wear one

Because it's hard to exercise while wearing a mask

**Video Stimuli**

Video 1: Control (Trains)

https://www.youtube.com/watch?v=ds6YpkI0mhc

Video 2: Science

https://www.youtube.com/watch?v=KTaMAgB00O4

Video 3: Liberty

https://www.youtube.com/watch?v=RQypElI6yeo

Video 4: Loyalty

https://www.youtube.com/watch?v=iqrwhwbvF14

Video 5: Combined

https://www.youtube.com/watch?v=5696fmQFxwI

**Text of Video Stimuli**

**VIDEO 1: CONTROL video (Trains):**

Trains are one of the worlds’ most important modes of transportation.

Today 40% of the world’s freight cargo is transported via trains.

The miles of railroad track in the United States reached its peak in 1916. That year there were more than 250,000 miles of track—enough to reach the moon from Earth.

The first steam-powered locomotives were built in England in the early 1800s.

In the United States, passenger trains carry more than 500 million riders per year.

The longest railway tunnel in the world is in Switzerland, and is more than 35 miles long.

The word “train” can be traced to a Latin word that means *pull* or *draw*.

Trains: the workhorses of the transportation system.

**VIDEO 2: SCIENCE video:**

According to the CDC, cloth coverings may slow the spread of the coronavirus, and prevent people who do not know they have it from transmitting it to others.

The coronavirus is spread largely through droplets that come out of the mouth and nose while talking, sneezing, coughing, or breathing. Even simple cloth face coverings can prevent these droplets from spreading.

This picture shows droplets exiting the mouth while speaking, with and without a face covering.

The CDC now recommends that everyone wear cloth face coverings when leaving their homes, regardless of whether they have fever or symptoms of COVID-19

Stanford Professor Larry Chu says: “As many as 40% of people infected with the virus that causes COVID-19 may have no symptoms. But when they talk, cough or sneeze, they can still spread the virus to others in the form of respiratory droplets expelled into the air. So, wearing a mask regularly can prevent spreading at the source even when we don’t know we are sick.”

A study that looked at coronavirus transmission rates across 198 countries found countries that introduced mask wearing earlier in the outbreak had drastically fewer deaths. According to the lead author of the study, “It wasn’t just by a few per cent, it was up to a hundred times less mortality.”

This chart shows deaths in countries that implemented mask-wearing compared to those that didn't.

Some people are concerned about masks trapping in carbon dioxide (CO2). According to Sarah Stanley, Professor of Infectious Diseases at Berkeley, there is no risk of CO2 poisoning or “hypercapnia” from wearing a mask. Remember, surgeons habitually wear masks for extended periods of time.

Recent reviews of the scientific literature and data models found that masks work best when everyone is wearing them. Science suggests masks are the most inexpensive way to curb the impact of the disease on our health and on the economy.

The evidence is clear. Wear a mask.

**VIDEO 3: LIBERTY**

America was founded on the principle that each individual has the right to life, liberty, and the pursuit of happiness.

Government-mandated lockdowns may slow the pandemic but they are an alarming suppression of freedom.

Suppressing freedom through lockdowns also hurts our economy. Without jobs, we have no livelihood.

There is a better way to slow the pandemic. And we can do it on our own, without big government, and with less impact on the economy.

The coronavirus is spread largely through droplets that come out of the mouth and nose while talking, sneezing, coughing, or breathing. Even simple cloth face coverings can prevent these droplets from spreading.

Masks allow us to safely leave our homes, to interact with each other. Masks allow us to do our work, to run our businesses, and to go about our lives.

Thanks to wide use of masks, countries like Japan, Singapore, and South Korea had little to no lockdowns or restrictions on their freedoms. They managed to prevent large outbreaks, keep death rates extremely low, and, most importantly, retained their freedom and went about their normal lives sooner.

We can take responsibility for ourselves and choose to wear masks, so that the government doesn’t resort to imposing another lockdown.

Choose freedom. Choose to wear a mask.

**VIDEO 4: LOYALTY**

Together, we can save America from the coronavirus and restore our nation’s economy.

A pandemic is not an individual’s disease. A pandemic endangers us all. It uses each one of us to spread to many others.

Thousands of Americans are getting sick and dying every day. The most vulnerable are of America’s greatest generations, the same Americans who fought for us in past wars.

The coronavirus is spread largely through droplets that come out of the mouth and nose while talking, sneezing, coughing, or breathing. Even simple cloth face coverings can prevent these droplets from spreading.

Masks can save American lives, and restore our nation’s economy, but only if everyone wears them.

In this time of national crisis, like our forefathers before us, it is our patriotic duty to come together and fight for our way of life, our traditions, and each other.

History has shown that when America is threatened, we rise as one.

Help protect America. Wear a mask.

**VIDEO 5: COMBINED**

(L) Together, we can save our nation from the coronavirus and restore America’s economy.

(L) Thousands of Americans are getting sick and dying every day., The most vulnerable are of America’s greatest generations, the same Americans who fought for us in past wars.

(F) Government-mandated lockdowns may slow the pandemic but they are an alarming suppression of freedom.

(F) There is a better way to slow the pandemic. And we can do it on our own, without big government, and with less impact on the economy.

The coronavirus is spread largely through droplets that come out of the mouth and nose while talking, sneezing, coughing, or breathing. Even simple cloth face coverings can prevent these droplets from spreading.

(S) This picture shows droplets exiting the mouth while speaking, with and without a face covering.

(S) A study that looked at coronavirus transmission rates across 198 countries found countries that introduced mask wearing earlier in the outbreak had drastically fewer deaths. According to the lead author of the study, “It wasn’t just by a few per cent, it was up to a hundred times less mortality.”

(S) This chart shows deaths in countries that implemented mask-wearing compared to those that didn't.

(F/L) Masks allow us to safely leave our homes, to interact with each other. Masks allow us to do our work, to run our businesses, and to go about our lives.

(S) Recent reviews of the scientific literature and data models found that masks work best when everyone is wearing them. Science suggests masks are the most inexpensive way to curb the impact of the disease on our health and on the economy.

(L) In this time of national crisis, like our forefathers before us, it is our patriotic duty to come together and fight for what we believe in, our way of life, our traditions, and each other.

(F) Thanks to wide use of masks, countries like Japan, Singapore, and South Korea had little to no lockdowns or restrictions on their freedoms. They managed to prevent large outbreaks, keep death rates extremely low, and, most importantly, retained their freedom and went about their normal lives sooner.

The evidence is clear. Choose freedom. Help protect America. Wear a mask.
